# Know Thyself: Behavioral Evidence for a Structural Representation of the Human Body

**DOI:** 10.1371/journal.pone.0005418

**Published:** 2009-05-01

**Authors:** Elena Rusconi, Mirandola Gonzaga, Michela Adriani, Christoph Braun, Patrick Haggard

**Affiliations:** 1 Institute of Cognitive Neuroscience, University College London, London, United Kingdom; 2 Center for Mind/Brain Sciences, University of Trento, Mattarello, Italy; 3 Department of Cognitive and Education Sciences, University of Trento, Rovereto, Italy; University of Sydney, Australia

## Abstract

**Background:**

Representing one's own body is often viewed as a basic form of self-awareness. However, little is known about structural representations of the body in the brain.

**Methods and Findings:**

We developed an inter-manual version of the classical “in-between” finger gnosis task: participants judged whether the number of untouched fingers between two touched fingers was the same on both hands, or different. We thereby dissociated structural knowledge about fingers, specifying their order and relative position within a hand, from tactile sensory codes. Judgments following stimulation on homologous fingers were consistently more accurate than trials with no or partial homology. Further experiments showed that structural representations are more enduring than purely sensory codes, are used even when number of fingers is irrelevant to the task, and moreover involve an allocentric representation of finger order, independent of hand posture.

**Conclusions:**

Our results suggest the existence of an allocentric representation of body structure at higher stages of the somatosensory processing pathway, in addition to primary sensory representation.

## Introduction

Knowledge about the physical structure of our own body is a basic form of self-awareness. We know that we have two arms, two eyes, ten fingers etc. However, there is no consensus, nor much systematic research, on how such representations of body structure are formed, and what information they contain. Primary somatosensory systems, notably touch, code basic features of sensory events at the skin, such as intensity and frequency. These are represented in the somatotopic map of primary somatosensory cortex, which allows stimuli to be localised on the continuous sheet of the body surface. However, these primary representations do not contain structural information about the size and number of body parts themselves. Moreover, the SI representation of the skin sheet does not respect the body's true proportions, and decays rapidly in less than 1 second in the absence of sensory input [1], [2]. These properties reflect the needs of online sensory processing, but make them unsuitable for representing the body itself.

Nevertheless, representation of body structure appears to be an important feature of higher processing in the somatosensory pathway. For example, tactile and proprioceptive inputs are segregated at early stages of somatosensory processing, in areas 3b, 1 and 2 respectively. However, cells in higher areas, notably SII and area 5 [3], [4], respond to both input modalities. By combining touch and proprioception, the brain can locate body parts in space, for example in order to orient towards a tactile stimulus [5], [6]. Thus, the brain updates the current position of body parts in egocentric space as they move. However, the brain also contains a second kind of spatial information, which we call *structural information*, about the location and size of body parts relative to one another. Examples of such information include, the number and order of the fingers, the positions of the joints within the limb, limbs on the trunk etc. The strongest evidence for these **Body Structural Representations** comes from neuropsychology. Thus, patients with autotopagnosia fail to point to specific body parts in response to instructions, yet can still use these body parts, and point towards them when they are stimulated. The pattern of errors often suggests that the patient has lost the ability to individuate body parts from each other, and organise them relative to each other: the command “point to your elbow” may produce vague pointing to the upper or lower part of the arm which cannot be explained by visuo-motor impairments [7], [8]. Autotopoagnosia also dissociates from more general impairments of visual mental imagery and is most frequently caused by left parietal damage [9], [10].

Finger agnosia is a more common and more restricted disturbance of body structural representation. These patients may have normal primary somatosensory processing, for example they may detect unseen touch on the fingers. However, they make errors in identifying *which* finger is stimulated. The errors mainly involve confusion between the central fingers on each hand, as if these had lost their individual identities and ordering. Patients make fewer errors when the stimulation is seen as well as felt, ruling out a mere confusion in using of finger names or labels. Rather, the deficit seems to be in connecting the primary somatosensory input to a structural description of the order and layout of body parts. Finally, these patients can order strips of paper labelled with the names of the fingers. Their verbal and semantic knowledge about finger identities and order is intact [11], but finger identity cannot be attached to somatosensory maps. In summary, individuation and ordering of fingers are not direct consequences of sensorimotor organisation, but involve specific mental operations. These have been localised to the left parietal lobes [12], although performance in finger gnosis tasks may also be impaired after right parietal damage putatively as a consequence of attentional deficits rather than body-specific impairments [13], [14]. Importantly, recent neuroimaging studies confirm the left lateralization. When healthy participants judged the distance between body parts the left intra-parietal area was activated, in a region clearly posterior to SI [15].

Here we investigate how somatosensory codes become connected to body structure representation (BSR), by testing knowledge about tactile stimulation of the fingers in the absence of vision. Fingers are a salient feature of human body structure, with rich sensory and motor innervation. The five digits are arranged in a clear, and normally fixed, sequential order [16]. We therefore performed four experiments reported aiming to dissociate structural knowledge about fingers from sensorimotor representations of finger stimuli and finger movements, and to reveal how tactile sensory input accesses the BSR.

Our main interest lay in dissociating sensory representations of touch stimuli from cognitive representations of body structure. Therefore, we used an intermanual version of the classical in-between task [12], in which participants were asked to say whether the distance between the two fingers touched on one hand was the same as the distance between the two fingers touched on the other hand. When the same fingers were touched on both hands (“total homology” trials), the answer ‘same’ could be given based on the homology of sensory representations alone, and the subject did not need to represent the structure of the hand in order to identify the untouched in-between fingers, nor compare structure between the hands. When the fingers touched on the two hands were not the same (“partial homology” and “no homology” trials), sensory representations are insufficient, and body structural representations must be used. We therefore compared the accuracy of performance as a function of the number of fingers in common on both hands.

In Experiment 2 we aimed to dissociate the time-courses of the sensory and structural representations involved in the intermanual in-between task by introducing an intermanual delay during stimulation. We reasoned that primary sensory codes have immediate onset, but decay rapidly [1]. Conversely, structural codes should take time to build up, but should then resist decay. On this reasoning, we predicted an interaction between delay and homology factors, with delay benefiting performance more (or impairing it less) for *no* homology and partial homology conditions than for total homology conditions.

Because the intermanual in-between task specifically refers to the number of digits on each hand, it is difficult to distinguish between the contributions of body structural description and numerical coding. Indeed, finger gnosis and number representation are generally strongly linked [12], [17]. To investigate whether our effects of homology could be due to numerical coding, rather than body representation, Experiment 3 compared the intermanual in-between task with an intensity judgement task using the same stimuli. In the intensity judgement task, participants judged whether the left or right hand received more intense stimulation: the identities of the fingers stimulated on each hand, and the number of fingers in between the fingers stimulated were irrelevant to the intensity task. If the intensity task showed the same effects of homology and delay as the intermanual in-between task, this would suggest that numerical coding is not responsible for the results found in the intermanual in-between task, and would strengthen the view that the putative secondary representations were somatic rather than simply numerical.

Experiment 4 sought to investigate the frame of reference used in the different trial types of the intermanual in-between task. The in-between task clearly involves spatial judgement about the distances between stimulated body parts. However, the nature of the spatial representation used remains unclear. There are at least 2 possibilities. First, touched locations could be remapped into egocentric spatial coordinates [6], and inter-digit distances computed as vectors between these egocentric co-ordinates. Alternatively, inter-digit distances could be computed in a representation of the hand which remains the same as the hand's position and posture in space are varied. Several studies show modulations of tactile perception when hand postures are incongruent [18] compared to when they are congruent. Such results are taken as evidence that individual tactile inputs are rapidly remapped according to hand posture in space. We therefore wanted to assess whether BSRs were modulated by postural congruence in the same way as individual tactile inputs.

## Materials and Methods

### Experiment 1 – The Intermanual In-Between Task

**Figure 1:**
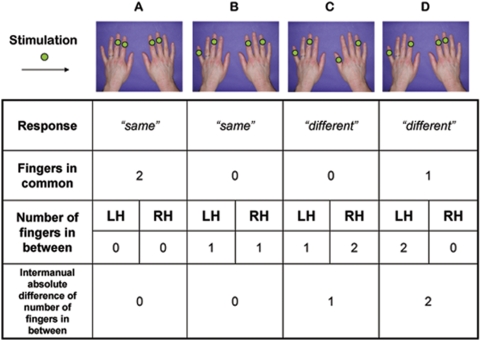
Example of stimuli and main variables of interest. Green dots on fingers indicate sites of stimulation. A No fingers in-between on either hand: a ‘same’ answer is required. Full homology is present, therefore this pattern is classified as a ‘total homology’ trial. B One finger in-between on either hand: a ‘same’ answer is required. No homology is present, therefore this pattern is classified as a ‘no homology’ trial. C One finger in-between on the left hand, two fingers in-between on the right hand: a ‘different’ answer is required. Inter-manual distance is given by the absolute difference between the number of fingers in-between on the two hands, and is equal to 1 for this pattern. D Two fingers in-between on the left hand, no fingers in-between on the right hand: a ‘different’ answer is required. Partial homology is present, since the index finger of both hands is stimulated, therefore this pattern is classified as a ‘partial homology’ trial.

Ten subjects (mean age: 20; range: 19–22; 10F) participated with the approval of UCL ethics committee. Subjects sat blindfold at a table, with their hands resting palms down on a computer monitor. Hand outlines on the monitor allowed the experimenter to standardise hand positions. On each trial, two digits on the left hand and two on the right hand were designated by colour patches at corresponding points on the monitor. The experimenter manually touched each of the four designated finger locations simultaneously, with a firm touch lasting around 1 s. Touch was applied to the dorsum of the distal phalanx, immediately proximal to the fingernail. Particular attention was paid to ensuring that all the four stimulated fingers were touched at the same time and with the same pressure. Subjects made unspeeded vocal responses to indicate whether the number of untouched fingers in between the two fingers touched was the same or different across the two hands. The task was effectively an intermanual two-alternative forced choice version of Kinsbourne and Warrington's [12] ‘in-between task’. Although there are more possible combinations (i.e. patterns of stimulation) requiring the response ‘different’ than the response ‘same’, we employed a selection of stimuli having ‘different’ as correct response, so that both responses were represented in a similar proportion within each block. We also ensured that the majority of possible finger pairs were represented. This allowed us to classify each trial according to the degree of homology, or number of fingers in common, to both hands. When the same fingers were touched on both hands, there was *total homology*, or 2 fingers in common between hands. When different fingers were touched on both hands, there was *no homology*, or no fingers in common between hands. When one particular finger was stimulated on both left and right hands, but the other fingers differed between the hands, there was *partial homology*, or 1 finger in common (Fig. 1). 256 trials were presented in pseudo-random order in 2 identical sessions of 128 trials, each divided in two blocks. The total homology trials were repeated across the two blocks (because of the limited number of patterns available), while no homology and partial homology trials presented in block 1 were structurally identical in the two blocks, but the allocation of stimulation to hands was reversed between blocks. Effects of repetition of identical items (which might have produced a spurious advantage for the total homology trials) were discarded by comparing performance between blocks. Since we found no effects or interactions involving block (Fs<1), we pooled data across blocks. Stimuli for each block and principle of classification are reported in Table S1.

### Experiment 2 – Intermanual Delay

12 new subjects (mean age: 23; range: 18–45; 10F) participated. Details were based on Experiment 1, except that in half the trials a delay of 3 s was inserted between stimulation onsets of the two hands. The order of stimulation was randomised. Subjects responded whether the number of untouched fingers in between was the same on the two hands, or not, as before. Since we wished subjects to retain a **bodily** representation during the delay, rather than immediately recoding the distance between the first two touched fingers into a purely numerical form, we also included 20% of randomly intermingled trials in which subjects were prompted whether any of the fingers touched was common to both hands. These ‘any same fingers?’ trials always involved either *no* or partial homology, so were never ambiguous (see Table S2). Each subject performed 324 trials, spread across 4 blocks, in pseudo-random order.

### Experiment 3 – Generalisation across tactile judgement tasks

6 subjects (mean age: 35; range: 28;51; 3F) took part with the permission of the University of Trento ethical committee. Stimuli were based on those for experiment 2. However, they were delivered by computer-controlled piezoelectric stimulators (Quaerosys, Stuttgart, Germany), rather than manually. Stimulators transducer voltage controlled displacements of a piezo crystal into protrusion or retraction of a plastic pin (ø 1 mm, maximal skin indentation 1.2 mm). The devices gave a brief buzz-like vibration to the stimulated digits (20 Hz). Stimulus intensity could be systematically varied by controlling the amplitude of the skin indentation. The same stimulus intensity was applied to the two fingers on each hand, but the stimuli were more intense on one hand than on the other. The experiment comprised two tasks. The intermanual in-between task used the same piezoelectric stimulation as the intensity task, but was otherwise designed as in Experiment 2. In this task, intensity was irrelevant. In a separate counterbalanced section of the experiment, participants judged which hand was more intensely stimulated. In this task, both the location of the fingers stimulated, and the number of untouched fingers in between was irrelevant. The two intensities of the experiment were chosen individually such that performance rates were similar in both tasks. Critically, in the intensity task, numerical coding would not facilitate performance. Moreover, the task was purely sensory in nature, and made no reference to body structural coding. After individually adjusting the difference between stimulation intensities to obtain the same level of accuracy in the two tasks, half the subjects performed the in-between task for 60 trials, followed by two blocks of 60 trials in which they performed the intensity task, and finally by another block of 60 trials in the in-between task. The other half was assigned the complementary order of tasks, always following an ABBA design.

### Experiment 4 – Effect of Hand Posture

**Figure 2:**
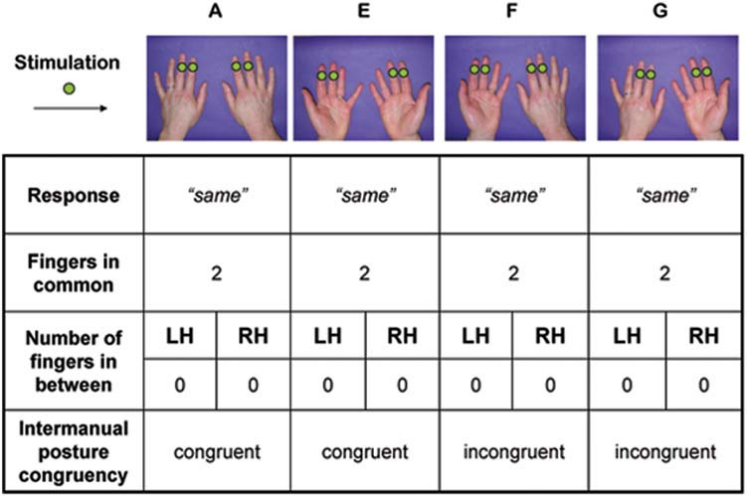
Example trials with postures adopted in Experiment 4. As in Figure 1, green dots on fingers indicate sites of stimulation. Panels A and E exemplify blocks in which both hands were kept by participants in a similar posture (either on their back or on their palm) and are therefore labelled as congruent condition. Panels F and G exemplify blocks in which one hand was on the palm and the other on the back and are therefore labelled as incongruent condition.

The design and procedure were largely based on Experiment 1. Sixteen new subjects (mean age: 26; range: 19–60; 10 female) participated, with the permission of UCL ethics committee, in four blocked conditions generated by orthogonally varying the posture of each hand to be palm-downwards or palm-upwards (Fig. 2). In the palm-downwards conditions, stimulation was as in experiment 1. In the palm-upwards conditions, stimulation was delivered to the distal phalanx, on the pad of the finger. Care was taken to keep the hand flat in the palm-up condition.

## Results

### Experiment 1 – The Intermanual In-Between Task

**Figure 3:**
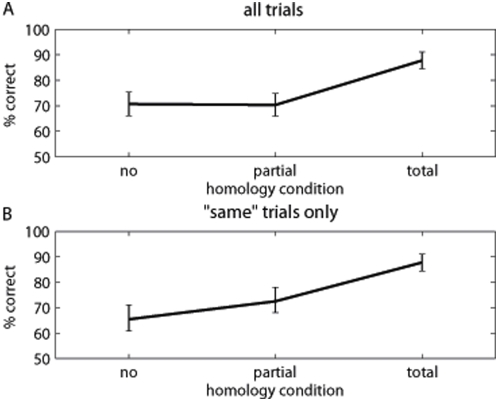
Mean percentage of correct responses for Experiment 1. Vertical bars indicate standard error of the mean. A Effect of homology over all trials (i.e. both ‘same’ and ‘different’ responses; with total homology, however, correct responses were always ‘same’). B Effect of homology for ‘same’ trials only.

A repeated measures ANOVA on percentage of accuracy with the factor homology (total, partial, none) revealed a significant effect of number of fingers in common (Fig. 3A: *F*(2,18) = 19.95, p<.0001, η^2^ = 689). Follow-up t-testing showed the total homology condition to differ significantly from partial homology (t(9) = 4.54, p = .0014, *d* = 1.435) and no homology (t(9) = 5.66, p = .0003, *d* = 1.791), which in turn did not differ significantly from each other (t<1). We reasoned that overall ANOVA might include a response bias, since total homology trials necessarily require the response ‘same’, while no homology and partial homology trials required the response ‘different’ 43% and 76% of the times respectively. We therefore analysed the subset of trials requiring the response ‘same’ in each condition. This showed a similar result, with total homology performance again significantly better than partial and *no* homology (Figure 3B: mean(se) 88%(3%), 73%(6%), and 65%(6%) respectively, *F*(2,18) = 24.02, p<.0001; total vs partial homology: t(9) = 4.11 p = .003, *d* = 1.298; total vs *no* homology: t(9) = 5.84, p = .0002, *d* = 1.847).

**Figure 4:**
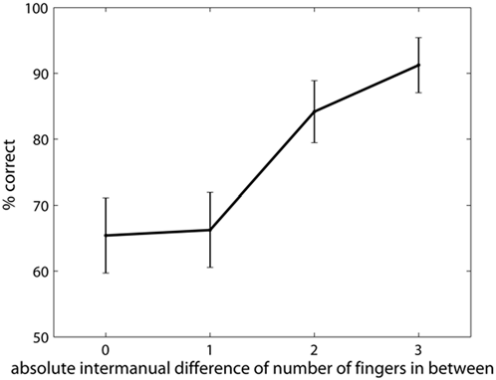
Response curve for the absolute inter-manual in-between difference. Vertical bars indicate standard error of the mean. Note that when the difference is zero, ‘same’ responses are required, when it is greater than zero, ‘different’ responses are required. A difference of 3 is given by a very limited number of patterns. Therefore only differences of 1 and 2 should be taken into account when testing the effect of inter-manual in-between distance.

The intermanual in-between task requires participants to compare information between the hemispheres, but does not reveal the nature of the information compared. Performance on *no* homology trials, however, cannot depend on sensory comparison alone. We investigated whether performance varied as a function of the *difference* in the number of in-between fingers on the two hands for the *no* homology trials alone. Note that this ‘difference value’ is independent of which actual fingers are touched, and (for difference values 1 and 2) is also independent of the number of fingers in between on each hand. For example, a difference value of 1 could arise from in-between values on the two hands of 0 and 1, 1 and 2, or 2 and 3). This showed a clear monotonic sigmoidal function, with worst performance when the numbers of in-between fingers were identical and best performance when the number of in-between fingers were maximally different (Figure 4: *F*(3,27) = 14.10, p<.0001, η^2^ = .689). We also performed a planned comparison restricted to difference values of 1 and 2, to give an indication of the perceptual dimension independent of specific stimulus and response values (difference values of 3 necessarily involve thumb and little finger stimulation, while difference values of 0 necessarily require the response ‘same’). This comparison was significant (t(9) = 3.11, p = 0.012, *d* = 0.982). This result suggests that some dimension of body structure specifying the number of in between fingers, but independent of any particular sensory input or response can be compared across hemispheres.

### Experiment 2 – Intermanual Delay

**Figure 5:**
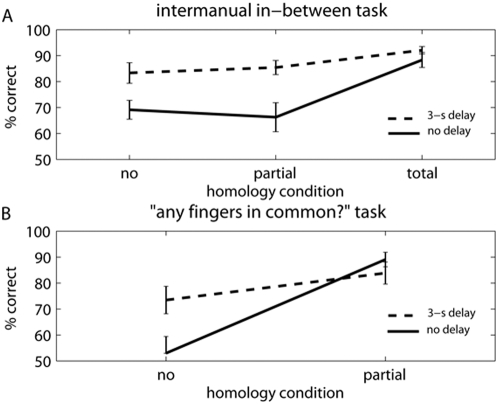
Mean percentage of correct responses for Experiment 2. Vertical bars indicate standard error of the mean. Continuous lines indicate trials in which touches were given simultaneously on both hands, dotted lines indicate trials in which a 3-s offset was present between touches on the two hands. A Effect of homology in the in-between task. B Effect of homology in the ‘any fingers in common?’ task.

The data from the in-between trials are shown in figure 5A, and were entered in a repeated measures ANOVA with homology (no, partial, total homology) and delay (no delay, 3-s delay) as factors. The effect of homology closely replicated Experiment 1 (*F*(2,22) = 12.29, p<.001, η^2^ = .524). Inter-manual delay significantly improved performance (*F*(1,11) = 32.90, p<.001, η^2^ = .749). Finally, there was a significant interaction (*F*(2,14) = 7.10, p = .014, η^2^ = .392; Greenhouse-Geisser corrected), with the benefit of delay being significant for partial and *no* homology trials (t(11) = 5.99, p<.0001, *d* = 1.684 and t(11) = 5.83, p = .0001, *d* = 1.730) but not for total homology trials (t<1). Performance on the ‘any fingers in common?’ trials is shown in figure 5B. There were significant effects of homology (F(1,11) = 25.36, p = .002, η^2^ = .697), of delay (F(1,11) = 14.67, p<.001, η^2^ = .571) and a significant interaction between homology and delay (F(1,11) = 5.78, p = .035, η^2^ = .586). The main effect of delay again showed that intermanual delays improved performance. The sensory codes required to identify whether there was no or partial homology must therefore have been retained over the delay period. Moreover, subjects could not predict whether a given stimulation would be followed by an ‘any common fingers’ judgement or an in-between judgement. Therefore, we can assume that subject did not simply discard sensory information, and retain only numerically recoded information about inter-finger distance. Information in a somatic form must have remained available in both tasks. The evidence from the two tasks together suggests that the in-between task involved either sensory or structural comparison, according to the number of fingers in common on the two hands.

### Experiment 3 – Generalisation across tactile judgement tasks

**Figure 6:**
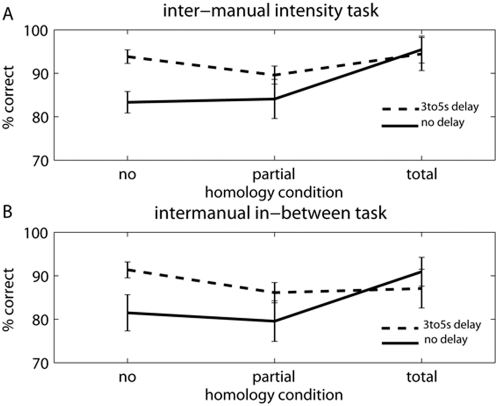
Mean percentage of correct responses for Experiment 3. Vertical bars indicate standard error of the mean). Continuous lines indicate trials in which touches were given simultaneously on both hands, dotted lines indicate trials in which a random 3-to-5-s offset was present between touches on the two hands. A Effect of homology in the intensity task. B Effect of homology in the in-between task.

The accuracy results were analysed using repeated measures ANOVA with factors of task (in-between, intensity), homology (none, total, partial), and delay (no delay, intermanual delay). The results followed the pattern of Experiments 1 and 2, with the familiar effects of homology (F(2,10) = 6.907, p = 0.014, η^2^ = .598), delay (F(1,5) = 9.302, p = 0.028, η^2^ = .650) and an interaction (F(2,10) = 5.541, p = 0.024, η^2^ = .529). There was no main effect of task (p>0.125). Critically, there was no hint of any interaction involving task (all Fs<1): the effects of homology and delay were similar in the intensity and in-between tasks (Fig. 6).

### Experiment 4 – Effect of Hand Posture

**Figure 7:**
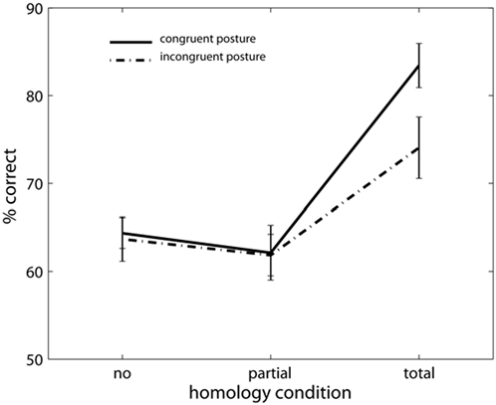
Mean percentage of correct responses for Experiment 4. Vertical bars indicate standard error of the mean). Continuous lines indicate trials in which hands were kept in an congruent posture, dotted lines indicate trials in which hands were kept in an incongruent posture. Patterns of touches were identical in the two conditions (i.e. the same combinations of fingers were stimulated, irrespective of posture).

The results are shown in figure 7. ANOVA revealed the familiar effect of number of fingers in common (*F*(1,15) = 34.23, p<.001, η^2^ = .695), a highly significant effect of congruence (*F*(1,15) = 14.00, p = .002, η^2^ = .483), and a significant interaction (*F*(1,15) = 4.74, p = .016, η^2^ = .241). Follow-up simple effect testing showed that the interaction occurred because total homology trials were modulated by the congruence of the two hands' postures while partial and no homology trials were not (total homology: t(15) = 3.40, p = .0039, *d* = .851; partial and no homology: ts<1).

## Discussion

The in-between task has a long history as a measure of body structure [12], [19]. A correct response requires firstly identifying which fingers are touched, and secondly locating the touched fingers within a structural model of the hand that represents at least the touched fingers and the untouched fingers in between them. In Experiment 1 our intermanual version allowed us to dissociate the sensory identification and structural representation components of the classic in-between task. Specifically, the structural component is unnecessary in the total homology condition, because sensory homology is sufficient to determine the response. We attribute the performance decrement in the partial and *no* homology conditions relative to total homology as reflecting the additional, presumably difficult, computations involving body structural representation. Interestingly, we found no difference between 1-common and 0-common conditions: when a BSR was required, the degree of sensory homology seemed irrelevant. This result is consistent with the sensory and structural stages of the task being independent [20].

The similar performance for *no* and partial homology trials also rules out explanations based on facilitation or interference between purely sensory codes. On that interpretation, performance in partial homology should lie between *no* homology and total homology conditions, since both facilitation and interference show classic “dose-response” relations. Our intermanual version also allowed us to investigate the principles of organisation of the BSR. Specifically, we found that the BSR contains information about the number of untouched in-between fingers. This information can be linked to current sensory input from the touched fingers, and can moreover be shared between the hemispheres.

In Experiment 2 an intermanual delay improved performance on our in-between task. We attribute this main effect to the difficulty of perceiving and localising several simultaneous touches. Tactile detection and pattern perception both fall off rapidly as the number of simultaneous touches increases above two [21], [22]. Our simultaneous condition required participants to localise four simultaneous touches, while the delayed condition required them to localise just two simultaneous touches. Delay thus improved performance by making the identification of each tactile pattern easier. Interestingly, however, this benefit was present only with partial and *no* homology trials and not with total homology trials. In other words, the representation used for partial and *no* homology conditions either decays less rapidly, or indeed becomes more elaborated through the delay interval, whereas the initial relative advantage of the total homology condition would be based on the immediate availability of primary somatosensory codes, which are sufficient to perform the task in that condition only. Such information, however, would also decay very rapidly, and the advantage of total homology disappears once participants cannot exploit any more the “somatosensory shortcut”. This fits the common observation that BSRs are relatively enduring, and outlast immediate sensory inputs. An alternative account might attribute the pattern of performance on total homology trials either to improved transcallosal facilitation in the simultaneous condition, or to slower decay of bilaterally homologous sensory codes in the delayed condition. However, such purely sensory accounts would again predict partial homology performance intermediate between the other conditions, which was not the case.

Experiment 3 showed that the detrimental effects of non-homology, and the beneficial effects of intermanual delay, were equally present for in-between and intensity comparisons. In principle, in-between tasks might be solved by simply converting structural information into a purely numerical code, and then comparing numerical values, without reference to body structure. However, this strategy would be of no value in judgements of a primary sensory dimension such as intensity. The pattern of results was nevertheless very similar for the two tasks. This makes it less plausible that the homology effects in the in-between task are due to a special task-specific coding strategy, such as number representation.

The intensity task of Experiment 3 also showed the hallmark effects of delay and homology. Thus, the characteristic effects associated with body structure representation coding were also present in purely implicit intensity task, which referred only to current stimulation, and did not refer explicitly to representation of untouched digits or position of stimulated digits within the hand. These results suggest that BSRs may be automatically activated by the tactile input, even when not necessary for the task. Simply judging attributes of tactile stimuli delivered to the fingers may automatically situate those stimuli within the body structure.

Alternatively, could the results of Experiment 3 be explained by a change in the neural representation of touch with increasing delays? Harris et al [1] asked participants to discriminate two vibration frequencies. The two vibrations could be delivered either to the same finger, or shifted by 1 or 2 fingers between the first and second vibration. The inter-stimulus interval (ISI) was varied. For short ISIs, the results showed a clear topographic tuning, with performance declining as the shift between stimuli increased from 0 to +1 to +2. When the ISI was increased, however, performance for +1 shifted stimuli increased to the same level as repeated stimulation of the same finger. This pattern of results is consistent with a gradual recoding of tactile information into a representation with a broader, less spatially-precise topographic organization, possibly corresponding to secondary somatosensory cortex. A similar recoding could, in principle contribute to the reduction in homology effects with delay in our intensity judgement task. However, we think this is unlikely for two reasons. First, in our task, participants are stimulated simultaneously on two fingers, whereas Harris' task involved stimulation of one digit at a time. The perceived intensity of stimulation to each hand therefore presumably *already* involves integrating over a broad region of somatotopic space, even before any intermanual comparison. Therefore, the normal retention of a single stimulus in high-acuity topographic cortex [23] over short intervals may play a lesser role in our task than in Harris et al's.. Second, we found no evidence that trials in which the same pattern was presented on both hands with a shift of 1 or 2 fingers showed any effects of shift size, nor any interaction between shift size and delay.

Experiment 4 confirmed a dissociation between spatial coding for total homology and other trials. The purely sensory codes assumed to underlie total homology performance were strongly affected by the congruence of the two hands' postures. This may seem paradoxical, since those sensory codes may correspond to early cortical representations such as the SI homunculus. However, several studies show that tactile detection and localisation are sensitive to external spatial aspects, such as hand position. The anatomical frame of reference of early sensory cortex is very rapidly recoded into external spatial coordinates [6] producing spatial congruence effects [18]. In contrast, the representations compared across the hands in partial and *no* homology were quite different, since they were independent of hand posture. In this sense, the BSR underlying partial and *no* homology judgements seems to be a representation of the hand that is independent of hand location and orientation. This suggests that the spatial organisation of the BSR is not only somatic (tied to specific body parts), but also allocentric (independent of the location and orientation of those parts).

In summary, we adapted a classic finger gnosis task, the in-between task, to investigate how information about body structure is represented and communicated between hemispheres. Subjects reported whether the number of untouched fingers in-between two touched fingers was the same on both hands, or not. The inter-manual version of the task allowed us to dissociate, for the first time, the sensory aspects of finger gnosis, i.e., localising the tactile stimulation within a primary sensory map, from the representational aspects, i.e., linking the stimulation to a structural representation of the hand. This representation would specify the number, order and arrangement of the fingers. In the classical, unimanual version of the task, these two processing stages cannot be clearly separated. We reliably found better inter-manual performance when homologous fingers were touched on both hands (‘total homology’ trials), compared to partial homology or no homology between hands. Further analyses suggested this was due to an additional computational stage representing number and order of fingers in-between, rather than mere overlap between sensory codes. We therefore proposed that a body structure representation (BSR) is used to compare tactile patterns across different body parts when sensory homology alone cannot be used. In further experiments, we confirmed the dissociation between sensory and structural representations on the basis of their time course (Experiment 2) and spatial frame of reference (Experiment 4). We also suggested that representation of the body used in the in-between task was also recruited for other intermanual sensory comparisons, in which digit representation is implicit and irrelevant (Experiment 3). The BSR was found to be long-lasting and posture-independent, relative to purely sensory representations.

What kind of information is processed in the intermanual in-between task? Clearly, at least a representation of body structure specifying the number of untouched fingers is necessary for the task. One might then ask whether the comparison between the two hands retains any body-specific content, or is a purely abstract numerical code. In Experiment 2, we showed that the characteristic pattern of homology effects remained even when rapid recoding into numerical form was discouraged by forcing participants to retain information about which specific fingers were touched for a randomly-interleaved task. Therefore, we suggest that an intermediate body-structural code is used between the original tactile sensation and the intermanual comparison. However, purely behavioural work cannot identify the precise nature of this code, or show how abstract it is. Future neuroimaging work might localize purely sensory tactile representations, and the abstract representations involved in numerical comparison, and then assess the degree of activation of each in the intermanual in-between task. Interestingly, body-structure and number codes seem to be closely associated in the brain. For example, Gerstmann's syndrome following left parietal damage involves both dyscalculia and finger agnosia. Therefore, our suggestion of an intermediate structural representation leading naturally to number codes may be consistent with the representational principles of the brain.

Our results provide a novel behavioural proof of the existence of a second level of body representation in the human brain. Studies of early sensory cortex reported somatotopic maps of tactile inputs [24]. Several areas of parietal association cortex rapidly remap these inputs into the external, egocentric spatial coordinates used for immediate motor control [25]. BSRs, in contrast, represent parts of the body that are not currently stimulated, and were dissociated from tactile sensory codes by our experimental manipulations. In particular, BSRs did not decay across time, were independent of hand posture, and appeared to be recruited automatically for intermanual comparison, even when not explicitly elicited by the task. In contrast, sensory representations are confined to the period of stimulation, and are linked to specific locations in somatotopic maps and in external space. Interestingly, spatio-temporal continuity is fundamental both to object constancy, and also to the sense of self. We suggest that the ability to form BSRs may represent an important cognitive and evolutionary step. A creature with a mind capable of BSR represents itself as a physical object with constancies like other physical objects. This may be an important precursor of self-awareness [26].

Our data also clarify several properties of the BSR. First, it can be compared across hemispheres. This implies an important cognitive operation of relational thinking or abstraction that is not captured by the classic in-between task. For example, someone who responds correctly to the stimulus of figure 1B must represent that the relation between fingers 2 and 4 (i.e., that they are separated by one in-between finger) is the same as the relation between fingers 3 and 5. The BSR therefore contains at least ordinal representations. BSRs also support the concept of two relations being similar even when the elements that are related are dissimilar, as in the example above. This relational quality represents an important stage of abstraction from purely sensory representations.

Finally, our behavioural results are strongly consistent with neuropsychological literature on body representation. Left hemisphere damage, notably to the angular gyrus, affects finger gnosis [12]. Our results show that, if the task is made sufficiently difficult, normal subjects make similar errors to patients. More generally, autotopagnosic patients show deficits in ordinal, magnitude and relational aspects of body representation. For example, when asked to point to their wrist, they may point to the shoulder or elbow [27]. On this evidence, Sirigu, Grafman, Bressler and Sunderland [28] proposed a structural representation specifying the spatial arrangement of different body parts relative to each other. Our data strongly supports this view, since the order of finger arrangement is a key structural feature of the hand. Crucially, our data shows the representation of body structure involves coding principles distinct from those used in early, sensory levels of somatosensory processing.

## Supporting Information

Table S1 All 100 experimental items are shown, 30 of which require a ‘same’ response, and 70 a ‘different’ response. Fingers are both number-coded. An ‘x’ in a column indicates that a tactile stimulus was delivered on the corresponding finger. Two experimental blocks were created with such items, one containing the 30 ‘same’ trials and half (i.e. 35) of the ‘different’ trials. Division of the ‘different’ trials in two halves was made in a way that configurations within each half were the mirror image of configurations in the other half. This was made to counterbalance the identity of stimulated fingers and overall difficulty between the two blocks of 65 trials (30 ‘same’ +35 ‘different’). In Experiment 1, each of the two blocks was repeated twice in a counterbalanced order, making up a total of 260 trials. Ibt L = number of fingers in between the stimulated fingers on the left hand; Ibt R = number of fingers in between the stimulated fingers on the right hand; Common = number of homologous fingers that are stimulated on the two hands. Supplementary Table 2 - The same structure as for Experiment 1 was maintained with the following exceptions: a) for half the items reported in Summary Table 1 stimulation was given simultaneously on the two hands, for the other half it was given with a delay of 3 s between hands (half the times starting with the left hand, the other half starting with the right hand); b) 32 additional trials were included in the experimental blocks (see below; 16 for each block that was repeated twice, as in Experiment 1, making up a total of 64 additional trials on the whole experiment), in which participants were asked to report whether the stimulated fingers were homologous or not. In half of these trials, stimulation occurred simultaneously on the two hands, in the other half it occurred with a 3-s delay between hands (half the times starting with the left hand).
(0.32 MB DOC)

Table S2 The same structure as for Experiment 1 was maintained with the following exceptions: a) for half the items reported in Summary Table 1 stimulation was given simultaneously on the two hands, for the other half it was given with a delay of 3 s between hands (half the times starting with the left hand, the other half starting with the right hand); b) 32 additional trials were included in the experimental blocks (see below; 16 for each block that was repeated twice, as in Experiment 1, making up a total of 64 additional trials on the whole experiment), in which participants were asked to report whether the stimulated fingers were homologous or not. In half of these trials, stimulation occurred simultaneously on the two hands, in the other half it occurred with a 3-s delay between hands (half the times starting with the left hand).
(0.12 MB DOC)
