# Supplementary material for: Know Thyself: Behavioral Evidence for a Structural Representation of the Human Body
Source: PLoS One. 2009 May 1;4(5):e5418. doi: 10.1371/journal.pone.0005418 (PMC2671600; doi:10.1371/journal.pone.0005418)
Supplement: Table S1 — All 100 experimental items are shown, 30 of which require a ‘same’ response, and 70 a ‘different’ response. Fingers are both number-coded. An ‘x’ in a column indicates that a tactile stimulus was delivered on the corresponding finger. Two experimental blocks were created with such items, one containing the 30 ‘same’ trials and half (i.e. 35) of the ‘different’ trials. Division of the ‘different’ trials in two halves was made in a way that configurations within each half were the mirror image of configurations in the other half. This was made to counterbalance the identity of stimulated fingers and overall difficulty between the two blocks of 65 trials (30 ‘same’ +35 ‘different’). In Experiment 1, each of the two blocks was repeated twice in a counterbalanced order, making up a total of 260 trials. Ibt L = number of fingers in between the stimulated fingers on the left hand; Ibt R = number of fingers in between the stimulated fingers on the right hand; Common = number of homologous fingers that are stimulated on the two hands. Supplementary Table 2 - The same structure as for Experiment 1 was maintained with the following exceptions: a) for half the items reported in Summary Table 1 stimulation was given simultaneously on the two hands, for the other half it was given with a delay of 3 s between hands (half the times starting with the left hand, the other half starting with the right hand); b) 32 additional trials were included in the experimental blocks (see below; 16 for each block that was repeated twice, as in Experiment 1, making up a total of 64 additional trials on the whole experiment), in which participants were asked to report whether the stimulated fingers were homologous or not. In half of these trials, stimulation occurred simultaneously on the two hands, in the other half it occurred with a 3-s delay between hands (half the times starting with the left hand). (0.32 MB DOC) [file pone.0005418.s001.doc]

**Table S1**

|  | **LEFT HAND** | | | | | **RIGHT HAND** | | | | |  |  |  |  |
| --- | --- | --- | --- | --- | --- | --- | --- | --- | --- | --- | --- | --- | --- | --- |
|  | **little** | **ring** | **middle** | **Index** | **thumb** | **Thumb** | **index** | **middle** | **ring** | **little** |  |  |  |  |
| **ITEM** | **5** | **4** | **3** | **2** | **1** | **1** | **2** | **3** | **4** | **5** | **response** | **in between LEFT** | **In between RIGHT** | **fingers in common** |
| 1 | x |  |  |  | x | x |  |  |  | x | same | 3 | 3 | 2 |
| 2 | x |  |  | x |  |  | x |  |  | x | same | 2 | 2 | 2 |
| 3 | x |  |  | x |  | x |  |  | x |  | same | 2 | 2 | 0 |
| 4 |  | x |  |  | x | x |  |  | x |  | same | 2 | 2 | 2 |
| 5 |  | x |  |  | x |  | x |  |  | x | same | 2 | 2 | 0 |
| 6 | x |  | x |  |  |  |  | x |  | x | same | 1 | 1 | 2 |
| 7 | x |  | x |  |  |  | x |  | x |  | same | 1 | 1 | 0 |
| 8 | x |  | x |  |  | x |  | x |  |  | same | 1 | 1 | 1 |
| 9 |  | x |  | x |  |  | x |  | x |  | same | 1 | 1 | 2 |
| 10 |  | x |  | x |  | x |  | x |  |  | same | 1 | 1 | 0 |
| 11 |  | x |  | x |  |  |  | x |  | x | same | 1 | 1 | 0 |
| 12 |  |  | x |  | x | x |  | x |  |  | same | 1 | 1 | 2 |
| 13 |  |  | x |  | x |  |  | x |  | x | same | 1 | 1 | 1 |
| 14 |  |  | x |  | x |  | x |  | x |  | same | 1 | 1 | 0 |
| 15 | x | x |  |  |  |  |  |  | x | x | same | 0 | 0 | 2 |
| 16 | x | x |  |  |  |  |  | x | x |  | same | 0 | 0 | 1 |
| 17 | x | x |  |  |  |  | x | x |  |  | same | 0 | 0 | 0 |
| 18 | x | x |  |  |  | x | x |  |  |  | same | 0 | 0 | 0 |
| 19 |  | x | x |  |  |  |  | x | x |  | same | 0 | 0 | 2 |
| 20 |  | x | x |  |  |  | x | x |  |  | same | 0 | 0 | 1 |
| 21 |  | x | x |  |  |  |  |  | x | x | same | 0 | 0 | 1 |
| 22 |  | x | x |  |  | x | x |  |  |  | same | 0 | 0 | 0 |
| 23 |  |  | x | x |  |  | x | x |  |  | same | 0 | 0 | 2 |
| 24 |  |  | x | x |  | x | x |  |  |  | same | 0 | 0 | 1 |
| 25 |  |  | x | x |  |  |  | x | x |  | same | 0 | 0 | 1 |
| 26 |  |  | x | x |  |  |  |  | x | x | same | 0 | 0 | 0 |
| 27 |  |  |  | x | x | x | x |  |  |  | same | 0 | 0 | 2 |
| 28 |  |  |  | x | x |  | x | x |  |  | same | 0 | 0 | 1 |
| 29 |  |  |  | x | x |  |  | x | x |  | same | 0 | 0 | 0 |
| 30 |  |  |  | x | x |  |  |  | x | x | same | 0 | 0 | 0 |
| 31 | x |  |  |  | x |  | x |  |  | x | different | 3 | 2 | 1 |
| 32 | x |  |  |  | x |  |  | x |  | x | different | 3 | 1 | 1 |
| 33 | x |  |  |  | x |  |  |  | x | x | different | 3 | 0 | 1 |
| 34 | x |  |  |  | x | x |  |  | x |  | different | 3 | 2 | 1 |
| 35 | x |  |  |  | x | x |  | x |  |  | different | 3 | 1 | 1 |
| 36 | x |  |  |  | x | x | x |  |  |  | different | 3 | 0 | 1 |
| 37 | x |  |  |  | x |  | x |  | x |  | different | 3 | 1 | 0 |
| 38 | x |  |  |  | x |  | x | x |  |  | different | 3 | 0 | 0 |
| 39 | x |  |  |  | x |  |  | x | x |  | different | 3 | 0 | 0 |
| **40** | **x** |  |  | **x** |  | **x** |  |  |  | **x** | **different** | **2** | **3** | **1** |
| 41 | x |  |  | x |  |  |  | x |  | x | different | 2 | 1 | 1 |
| 42 | x |  |  | x |  |  |  |  | x | x | different | 2 | 0 | 1 |
| 43 | x |  |  | x |  | x | x |  |  |  | different | 2 | 0 | 1 |
| 44 | x |  |  | x |  |  | x | x |  |  | different | 2 | 0 | 1 |
| 45 | x |  |  | x |  |  | x |  | x |  | different | 2 | 1 | 1 |
| 46 | x |  |  | x |  |  |  | x | x |  | different | 2 | 0 | 0 |
| 47 | x |  |  | x |  | x |  | x |  |  | different | 2 | 1 | 0 |
| 48 |  | x |  |  | x | x | x |  |  |  | different | 2 | 0 | 1 |
| 49 |  | x |  |  | x | x |  | x |  |  | different | 2 | 1 | 1 |
| **50** |  | **x** |  |  | **x** | **x** |  |  |  | **x** | **different** | **2** | **3** | **1** |
| 51 |  | x |  |  | x |  | x |  | x |  | different | 2 | 1 | 1 |
| 52 |  | x |  |  | x |  |  | x | x |  | different | 2 | 0 | 1 |
| 53 |  | x |  |  | x |  |  |  | x | x | different | 2 | 0 | 1 |
| 54 |  | x |  |  | x |  | x | x |  |  | different | 2 | 0 | 0 |
| 55 |  | x |  |  | x |  |  | x |  | x | different | 2 | 1 | 0 |
| **56** | **x** |  | **x** |  |  |  | **x** |  |  | **x** | **different** | **1** | **2** | **1** |
| 57 | x |  | x |  |  |  | x | x |  |  | different | 1 | 0 | 1 |
| 58 | x |  | x |  |  |  |  | x | x |  | different | 1 | 0 | 1 |
| 59 | x |  | x |  |  |  |  |  | x | x | different | 1 | 0 | 1 |
| **60** | **x** |  | **x** |  |  | **x** |  |  | **x** |  | **different** | **1** | **2** | **0** |
| 61 | x |  | x |  |  | x | x |  |  |  | different | 1 | 0 | 0 |
| **62** | **x** |  | **x** |  |  | **x** |  |  |  | **x** | **different** | **1** | **3** | **1** |
| **63** |  | **x** |  | **x** |  | **x** |  |  |  | **x** | **different** | **1** | **3** | **0** |
| 64 |  | x |  | x |  |  |  |  | x | x | different | 1 | 0 | 1 |
| 65 |  | x |  | x |  |  |  | x | x |  | different | 1 | 0 | 1 |
| **66** |  | **x** |  | **x** |  | **x** |  |  | **x** |  | **different** | **1** | **2** | **1** |
| 67 |  | x |  | x |  | x | x |  |  |  | different | 1 | 0 | 1 |
| **68** |  | **x** |  | **x** |  |  | **x** |  |  | **x** | **different** | **1** | **2** | **1** |
| 69 |  | x |  | x |  |  | x | x |  |  | different | 1 | 0 | 1 |
| 70 |  |  | x |  | x | x | x |  |  |  | different | 1 | 0 | 1 |
| **71** |  |  | **x** |  | **x** | **x** |  |  | **x** |  | **different** | **1** | **2** | **1** |
| **72** |  |  | **x** |  | **x** | **x** |  |  |  | **x** | **different** | **1** | **3** | **1** |
| 73 |  |  | x |  | x |  | x | x |  |  | different | 1 | 0 | 1 |
| 74 |  |  | x |  | x |  |  | x | x |  | different | 1 | 0 | 1 |
| **75** |  |  | **x** |  | **x** |  | **x** |  |  | **x** | **different** | **1** | **2** | **0** |
| 76 |  |  | x |  | x |  |  |  | x | x | different | 1 | 0 | 0 |
| **77** | **x** | **x** |  |  |  |  |  | **x** |  | **x** | **different** | **0** | **1** | **1** |
| **78** | **x** | **x** |  |  |  |  | **x** |  |  | **x** | **different** | **0** | **2** | **1** |
| **79** | **x** | **x** |  |  |  | **x** |  |  |  | **x** | **different** | **0** | **3** | **1** |
| **80** | **x** | **x** |  |  |  |  | **x** |  | **x** |  | **different** | **0** | **1** | **1** |
| **81** | **x** | **x** |  |  |  | **x** |  |  | **x** |  | **different** | **0** | **2** | **1** |
| **82** | **x** | **x** |  |  |  | **x** |  | **x** |  |  | **different** | **0** | **1** | **0** |
| **83** |  | **x** | **x** |  |  |  | **x** |  | **x** |  | **different** | **0** | **1** | **1** |
| **84** |  | **x** | **x** |  |  | **x** |  |  | **x** |  | **different** | **0** | **2** | **1** |
| **85** |  | **x** | **x** |  |  |  |  | **x** |  | **x** | **different** | **0** | **1** | **1** |
| **86** |  | **x** | **x** |  |  | **x** |  | **x** |  |  | **different** | **0** | **1** | **1** |
| **87** |  | **x** | **x** |  |  | **x** |  |  |  | **x** | **different** | **0** | **3** | **0** |
| **88** |  | **x** | **x** |  |  |  | **x** |  |  | **x** | **different** | **0** | **2** | **0** |
| **89** |  |  | **x** | **x** |  |  | **x** |  | **x** |  | **different** | **0** | **1** | **1** |
| **90** |  |  | **x** | **x** |  |  | **x** |  |  | **x** | **different** | **0** | **2** | **1** |
| **91** |  |  | **x** | **x** |  | **x** |  | **x** |  |  | **different** | **0** | **1** | **1** |
| **92** |  |  | **x** | **x** |  |  |  | **x** |  | **x** | **different** | **0** | **1** | **1** |
| **93** |  |  | **x** | **x** |  | **x** |  |  | **x** |  | **different** | **0** | **2** | **0** |
| **94** |  |  | **x** | **x** |  | **x** |  |  |  | **x** | **different** | **0** | **3** | **0** |
| **95** |  |  |  | **x** | **x** | **x** |  | **x** |  |  | **different** | **0** | **1** | **1** |
| **96** |  |  |  | **x** | **x** | **x** |  |  |  | **x** | **different** | **0** | **3** | **1** |
| **97** |  |  |  | **x** | **x** | **x** |  |  | **x** |  | **different** | **0** | **2** | **1** |
| **98** |  |  |  | **x** | **x** |  | **x** |  | **x** |  | **different** | **0** | **1** | **1** |
| **99** |  |  |  | **x** | **x** |  | **x** |  |  | **x** | **different** | **0** | **2** | **1** |
| **100** |  |  |  | **x** | **x** |  |  | **x** |  | **x** | **different** | **0** | **1** | **0** |
