# Supplementary material for: Know Thyself: Behavioral Evidence for a Structural Representation of the Human Body
Source: PLoS One. 2009 May 1;4(5):e5418. doi: 10.1371/journal.pone.0005418 (PMC2671600; doi:10.1371/journal.pone.0005418)
Supplement: Table S2 — The same structure as for Experiment 1 was maintained with the following exceptions: a) for half the items reported in Summary Table 1 stimulation was given simultaneously on the two hands, for the other half it was given with a delay of 3 s between hands (half the times starting with the left hand, the other half starting with the right hand); b) 32 additional trials were included in the experimental blocks (see below; 16 for each block that was repeated twice, as in Experiment 1, making up a total of 64 additional trials on the whole experiment), in which participants were asked to report whether the stimulated fingers were homologous or not. In half of these trials, stimulation occurred simultaneously on the two hands, in the other half it occurred with a 3-s delay between hands (half the times starting with the left hand). (0.12 MB DOC) [file pone.0005418.s002.doc]

**Table S2**

|  | **LEFT HAND** | | | | | **RIGHT HAND** | | | | |  |  |  |  |
| --- | --- | --- | --- | --- | --- | --- | --- | --- | --- | --- | --- | --- | --- | --- |
|  | **little** | **ring** | **middle** | **Index** | **thumb** | **thumb** | **index** | **middle** | **ring** | **little** |  |  |  |  |
| **NEW ITEM** | **5** | **4** | **3** | **2** | **1** | **1** | **2** | **3** | **4** | **5** | **response** | **in between LEFT** | **in between RIGHT** | **fingers in common** |
| 1a |  | x |  | x |  | x |  | x |  |  | No | 1 | 1 | 0 |
| 2a | x |  |  | x |  | x |  |  | x |  | No | 2 | 2 | 0 |
| 3a |  | x | x |  |  | x | x |  |  |  | No | 0 | 0 | 0 |
| 4a | x |  | x |  |  |  | x |  | x |  | No | 1 | 1 | 0 |
| 5a |  | x |  |  | x |  |  | x |  | x | No | 2 | 1 | 0 |
| 6a |  | x |  |  | x |  | x |  |  | x | No | 2 | 2 | 0 |
| 7a | x |  |  |  | x |  |  | x | x |  | No | 3 | 0 | 0 |
| 8a | x | x |  |  |  |  | x | x |  |  | No | 0 | 0 | 0 |
| 9a |  | x |  | x |  |  |  |  | x | x | Yes | 1 | 0 | 1 |
| 10a | x |  |  | x |  |  | x |  | x |  | Yes | 2 | 1 | 1 |
| 11a |  | x |  |  | x |  |  | x | x |  | Yes | 2 | 0 | 1 |
| 12a | x |  |  |  | x | x |  |  | x |  | Yes | 3 | 2 | 1 |
| 13a | x |  |  |  | x | x |  | x |  |  | Yes | 3 | 1 | 1 |
| 14a |  |  | x |  | x |  |  | x |  | x | Yes | 1 | 1 | 1 |
| 15a | x |  | x |  |  | x |  | x |  |  | Yes | 1 | 1 | 1 |
| 16a |  | x | x |  |  |  |  |  | x | x | Yes | 0 | 0 | 1 |
| 1b |  | x |  |  | x |  | x |  |  | x | No | 2 | 2 | 0 |
| 2b |  |  | x |  | x |  | x |  | x |  | No | 1 | 1 | 0 |
| 3b |  | x |  | x |  |  |  | x |  | x | No | 1 | 1 | 0 |
| 4b |  |  | x |  | x |  | x |  |  | x | No | 1 | 2 | 0 |
| 5b |  | x | x |  |  |  | x |  |  | x | No | 0 | 2 | 0 |
| 6b | x |  | x |  |  | x |  |  | x |  | No | 1 | 2 | 0 |
| 7b | x | x |  |  |  | x |  | x |  |  | No | 0 | 1 | 0 |
| 8b | x |  | x |  |  | x |  | x |  |  | Yes | 1 | 1 | 1 |
| 9b |  |  | x |  | x |  |  | x |  | x | Yes | 1 | 1 | 1 |
| 10b | x | x |  |  |  |  |  | x | x |  | Yes | 0 | 0 | 1 |
| 11b |  |  |  | x | x |  | x |  |  | x | Yes | 0 | 2 | 1 |
| 12b |  | x | x |  |  |  | x | x |  |  | Yes | 0 | 0 | 1 |
| 13b |  | x | x |  |  |  |  |  | x | x | Yes | 0 | 0 | 1 |
| 14b |  |  | x |  | x | x |  |  |  | x | Yes | 1 | 3 | 1 |
| 15b | x |  | x |  |  |  | x |  |  | x | Yes | 1 | 2 | 1 |
| 16b |  | x | x |  |  |  | x |  | x |  | Yes | 0 | 1 | 1 |
